# Prospective randomized comparison of cerebrospinal fluid aspiration and conventional popping methods using 27-gauge spinal needles in patients undergoing spinal anaesthesia

**DOI:** 10.1186/s12871-020-0954-9

**Published:** 2020-01-30

**Authors:** J. U. Han, B. G. Kim, C. Yang, W. H. Choi, J. Jeong, K. J. Lee, H. Kim

**Affiliations:** Department of Anesthesiology and Pain Medicine, Inha University College of Medicine, 27, Inhang-ro, Jung-gu, Incheon, Republic of Korea

**Keywords:** Aspiration, Cerebrospinal fluid, Lumbar puncture, Spinal anaesthesia

## Abstract

**Background:**

Performing spinal anaesthesia using the conventional popping method with a 27-gauge (27G) spinal needle is technically difficult. In this study, we compared the aspiration and conventional popping method for spinal anaesthesia using 27G Quincke-type needles.

**Methods:**

This prospective, randomized study enrolled 90 patients, aged 19 to 65 years, with American Society of Anesthesiologists physical status I-III, who were undergoing spinal anaesthesia. Patients were randomly assigned to one of two groups using a computer-generated random number table: patients receiving spinal anaesthesia using the aspiration method, in which the needle is advanced with continuous aspiration, or the conventional popping method. The primary outcome measure was the success rate of the first attempt to perform dural puncture. Number of attempts and passages, withdrawal cases, successful attempt time, total procedure time, and actual depth of dural puncture were recorded.

**Results:**

Eighty-eight patients were included in the study. In the aspiration group, the success rate of first attempt for dural puncture was 93.3%, compared with 72.1% in the popping group (*P* = 0.019). Success involving needle withdrawal was recorded in 4 (8.9%) patients in the aspiration group and 13 (30.2%) in the popping group (*P* = 0.024). In the popping group, the number of attempts was significantly higher (*P* = 0.044), and total procedure time was significantly longer (*P* = 0.023). Actual depths of dural puncture were deeper in the popping group than in the aspiration group (*P* = 0.019).

**Conclusions:**

The aspiration method using a 27G Quincke-type needle offers clinical benefits for dural puncture compared with the conventional popping method for spinal anaesthesia.

**Trial registration:**

Clinical research information service number: KCT0002815, registered 21/Apr/2018. Retrospectively registered.

## Background

Spinal anaesthesia has various advantages and has been widely used for anaesthesia of the lower abdomen, genitourinary organs, and lower extremity. However, technique-related adverse effects have been reported due to spinal anaesthesia, such as backache, post-dural puncture headache (PDPH), and transient neurological symptoms. The appropriate external diameter of the spinal anaesthesia needle is important to prevent these mechanical complications. Notably, PDPH is a common and troublesome complication; however, it is less likely to occur when using a thinner spinal needle [1, 2]. Therefore, efforts have been made to use thinner needles, with punctures largely being performed with a 27-gauge (27G) needle in most patients.

Using a thinner needle for spinal anaesthesia has several advantages. However, this procedure is more difficult to perform and prolonged [3–5]. A thinner needle is more flexible, which restricts advancement of the needle in the intended direction [6–8]. Additionally, the practitioner cannot feel the dural puncture (known as the dural click or popping sensation), which can lead to a higher failure rate when performing spinal anesthesia [9]. In addition, nerve damage or vascular injury may be caused by inserting the spinal needle too deep, due to uncertainty as to whether the dural puncture occurred.

In our institute, a new method has been used in > 10,000 cases over the past 10 years to advance the spinal needle with continuous aspiration into the syringe, by using a 27G needle. Here, we hypothesized that the aspiration method will provide more effective and safer spinal anaesthesia, compared with the conventional method. Therefore, the aim of this study was to compare the success rate, procedure time, depth of dural puncture, and complications between the aspiration method and the conventional popping method, using a 27G Quincke-type needle in adult patients undergoing spinal anaesthesia.

## Methods

This study adheres to CONSORT guidelines and was registered with the Clinical Research Information Service (identifier: KCT0002815) after the protocol was approved by the Institutional Review Board of Inha University Hospital (Incheon, Republic of Korea). Written informed consent was obtained from adults undergoing elective or emergency lower abdominal or lower limb surgery under spinal anaesthesia. Ninety patients, 19 to 65 years of age, with of American Society of Anesthesiologists physical status I-III, and who fulfilled the study inclusion criteria were recruited. Patients with any contraindications to spinal anaesthesia (increased intracranial pressure, infection on injection site, coagulation disorder, severe hypovolemia, and aortic and/or mitral stenosis), body mass index > 40 kg·(m^2^)^− 1^, history of spinal surgery, or congenital spinal deformity, were excluded. The patients were randomly assigned to one of two groups using a computer-generated random number table: patients in the popping group underwent spinal anaesthesia using the conventional popping method; those in the aspiration group received spinal anaesthesia using the novel aspiration method.

On entering the operating room, patients were monitored using electrocardiogram, pulse oximetry, and non-invasive blood pressure monitoring, measured every 5 min. After preloading with 5 ml·kg^− 1^ of crystalloid, the patients were placed in the lateral decubitus position, on their side with their knees flexed and pulled high against the abdomen or chest, assuming a foetal position. All procedures were performed by a single expert anaesthesiologist. The operator had 3 years of experience in both the conventional popping and aspiration methods for spinal anaesthesia.

When lumbar spine X-rays were taken of the patients, the expected depth was measured as follows. We defined the junction of the inferior vertebral notch and inferior articular facet of the L3 vertebrate as the point of intersection with the posterior dura complex. The operator draws a line connecting the posterior dura complex and the nearest skin point, then extends the line to the L3 vertebral body on the lumbar spine lateral view. We defined the contact point of the line and the L3 vertebral body as the anterior dura complex. Before spinal anaesthesia, the operator measured distances from the skin to posterior dura complex, and from the skin to anterior dura complex.

After disinfection of the patient’s back and skin, injection of local anaesthetic (2 ml lidocaine 2%) at L3-L4 was performed; spinal anaesthesia was performed using the 27G Quincke-type needle (spinal needle with Quinke bevel, Tachang Industrial co., Gongju, South Korea) and a 22G introducer needle attached to a 5 ml syringe (BD Emerald™ Syringe Luer Slip, Becton Dickinson India Pvt. Ltd., Rewari, India). The needle was inserted horizontally using the midline approach in all cases. In the aspiration group, cued by the sensation that the needle passed through the ligamentum flavum and subcutaneous tissue at the discretion of the operator, the stylet was removed. A 3-ml syringe (BD Emerald™ Syringe Luer Slip, Becton Dickinson India Pvt. Ltd.) was then connected to the needle and advanced while continuing aspiration with slight negative pressure till the cerebrospinal fluid (CSF) reached the syringe hub. In the popping group, the operator performed the conventional method, advancing the needle until the dural click was recognized. CSF was confirmed after the stylet was removed when CSF flowed out of the spinal needle. If the operator decided that the needle was too deep, considering the lumbar spine lateral view, or was contacting the bone, the needle was withdrawn until the subcutaneous tissue while checked the flow of CSF without the stylet without the stylet in the both groups. If CSF was present while withdrawing the needle, it was defined as a withdrawal case. This process of moving the spinal needle forward and backward movement was defined as a passage. If the operator could not confirm CSF flow during needle withdrawal, the needle was advanced repeatedly while changing its direction. If the operator could not confirm CSF flow during passages after 5 times, the attempt was regarded to be a failure. The needle was then completely removed and reinserted. If the operator could not confirm CSF flow even after 3 attempts, it was considered to be a failed case and the patient was excluded from the study. After confirming CSF flow, hyperbaric bupivacaine (Marcaine 0.5% Spinal Heavy®, AstraZeneca AB, Sodertalje, Sweden) was injected according to the patient’s height and surgical indication. Before removing the needle after injection of the drug, the operator placed sterile tape on the needle at the point where it met the skin. Sensory block was determined using an alcohol stick 10 min after administration of the local anaesthetics.

Another anaesthesiologist recorded the number of attempts, number of passages, withdrawal cases, successful attempt time, total procedure time, and complications. The primary outcome measure was the success rate of the first attempt on the dural puncture procedure. The successful attempt time was defined as the time from insertion of the spinal needle into the introducing needle to identification of CSF during the successful attempt. The total procedure time was defined as the time from the initial spinal needle insertion into the introducing needle to identification of the CSF. The distances from skin to anterior or posterior dura mater from the lumbar spine lateral view were recorded. The actual depth of dural puncture was defined as the needle depth into the back of the patient at time of CSF identification. Doses of hyperbaric bupivacaine and levels of spinal anaesthesia used in both groups were recorded. Complications, including paraesthesia, and bloody tap were also recorded. The enrolled patients were followed up until discharge to assess the occurrence of PDPH.

### Statistical analysis

The sample size estimations were performed in accordance with data from a pilot study, in which the success rate of first attempt was compared between the conventional popping method (45%) and aspiration method (82%) in 22 patients. We estimated that 90 subjects would be required to provide 95% power at a 5% significance level, which accounts for a 15% loss of study participants. Statistical analyses were performed using SPSS 19.0 (SPSS Inc., Chicago, IL, USA). All data are expressed as mean (standard deviation), number (%), or median (interquartile range [IQR]), as indicated. After testing for normally distributed data using the Kolmogorov-Smirnov test and Shapiro-Wilk test, continuous and categorical variables were analysed using a two-sample t-test and a chi-squared test, respectively. The variables that did not show normal distribution were analysed using a Mann-Whitney U test. Statistical significance was defined as *P* < 0.05.

## Results

**Fig. 1:**
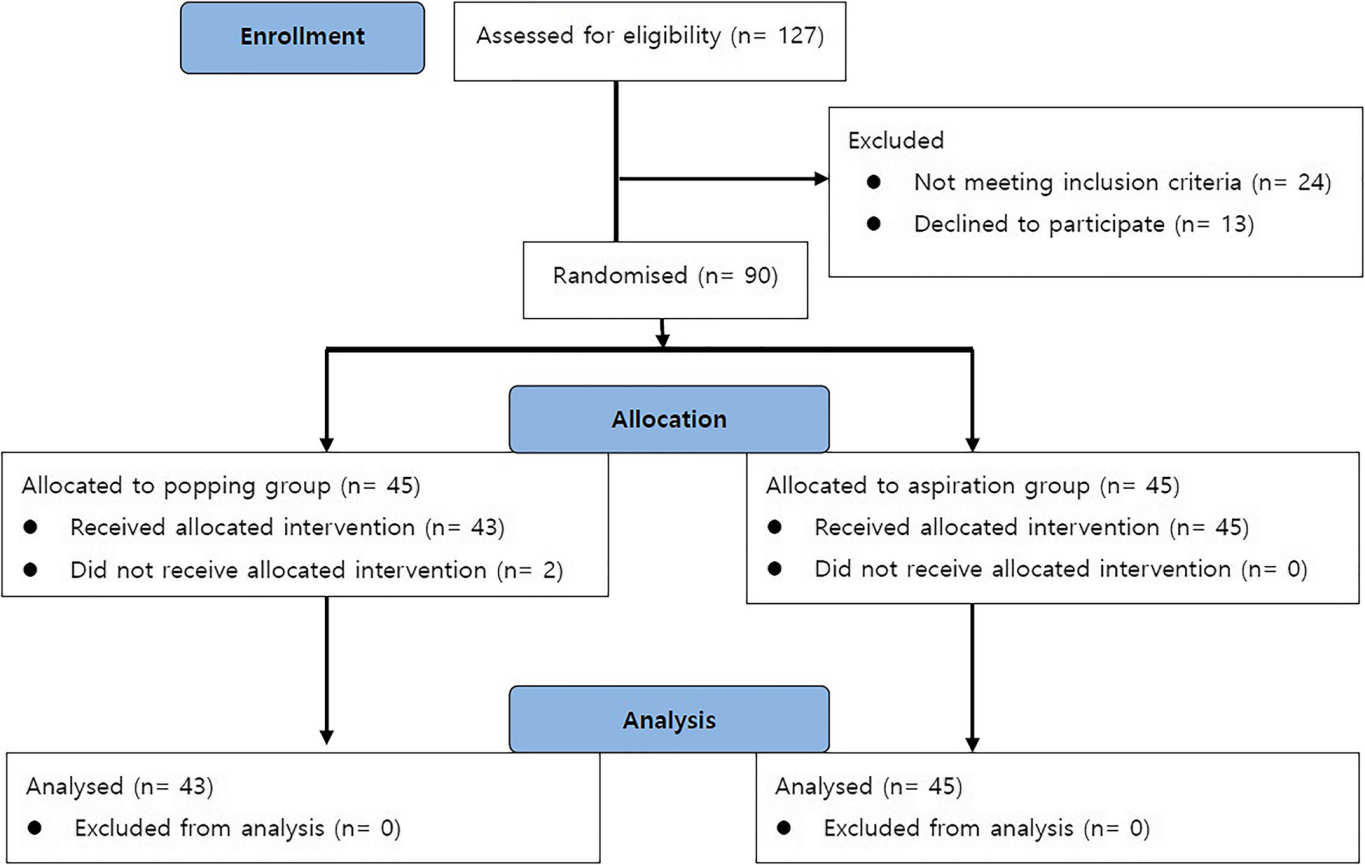
Enrollment and allocation of patients in the study

Fig. 1 illustrates patient enrolment and flow in the study. Of the 90 patients enrolled in the present study, two patients in the popping group were excluded because dural puncture failed. Therefore, 88 patients were included in the final analysis. There were no statistically significant differences in patient characteristics between the two groups (Table 1). The distances from skin to anterior or posterior dura mater in lumbar spine lateral view were comparable in both groups.

**Table 1:** Demographic data of participants undergoing aspiration or conventional popping for spinal anesthesia

Characteristic	Dural puncture		*P* value
	Popping group (*n* = 43)	Aspiration group (*n* = 45)	
Age (years)	44.3 ± 12.8	42.9 ± 14.0	0.623
Sex, male/female (n/n)	26/17	33/12	0.293
Height (cm)	165.5 ± 9.9	167.2 ± 7.3	0.369
Weight (kg)	68.9 ± 11.8	67.9 ± 11.2	0.672
Body mass index (kg/m^2^)	25.1 ± 3.8	24.2 ± 3.5	0.291
Distance to posterior dura mater (mm)	6.5 ± 1.3	6.4 ± 1.5	0.409
Distance to anterior dura mater (mm)	7.7 ± 1.2	7.6 ± 1.3	0.533

Data are presented as mean ± standard deviation unless otherwise indicated

**Fig. 2:**
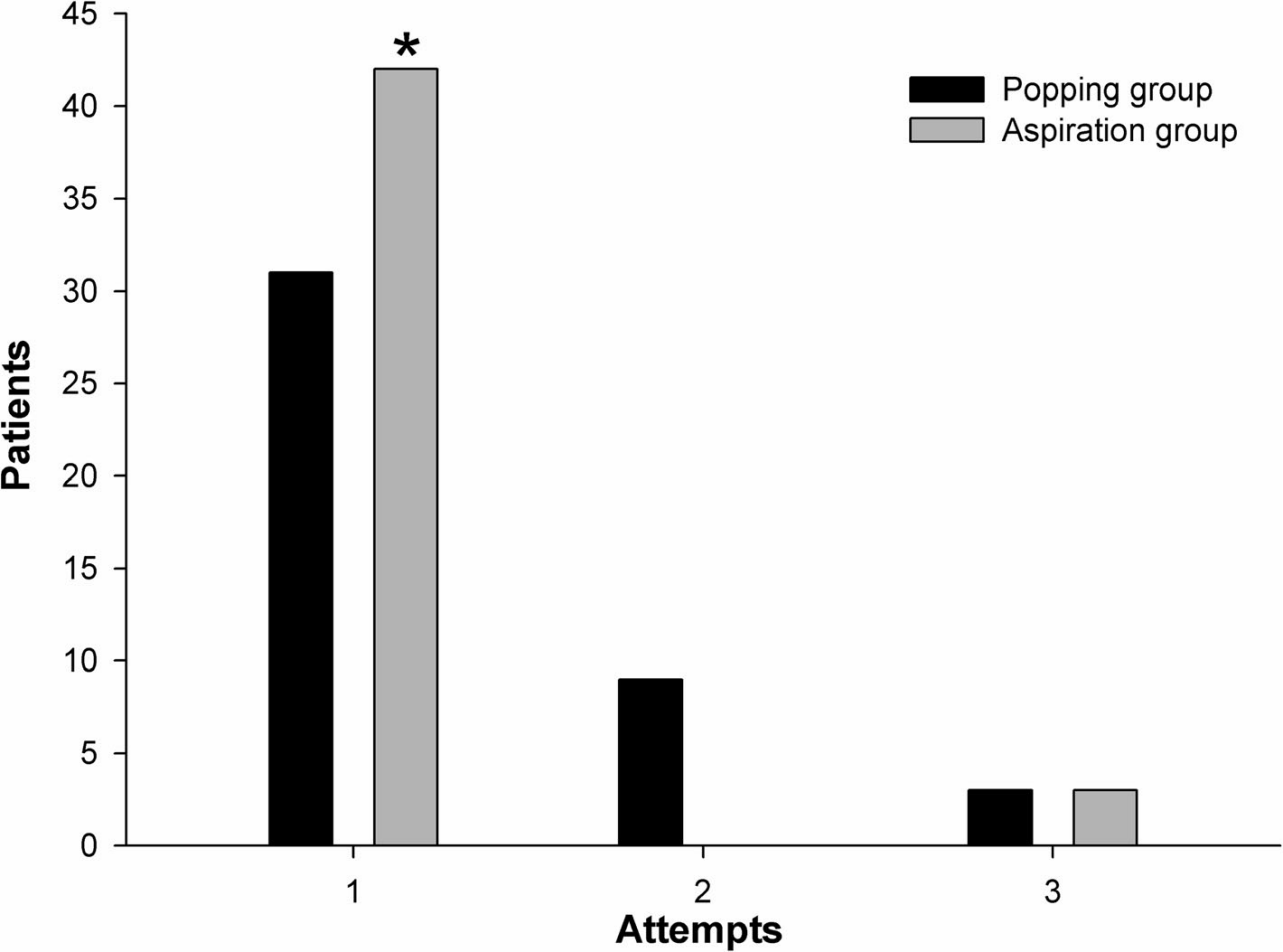
Number of attempts to achieve successful dural puncture in both groups. *Statistically significant (*P* ≤ 0.05)

**Fig. 3:**
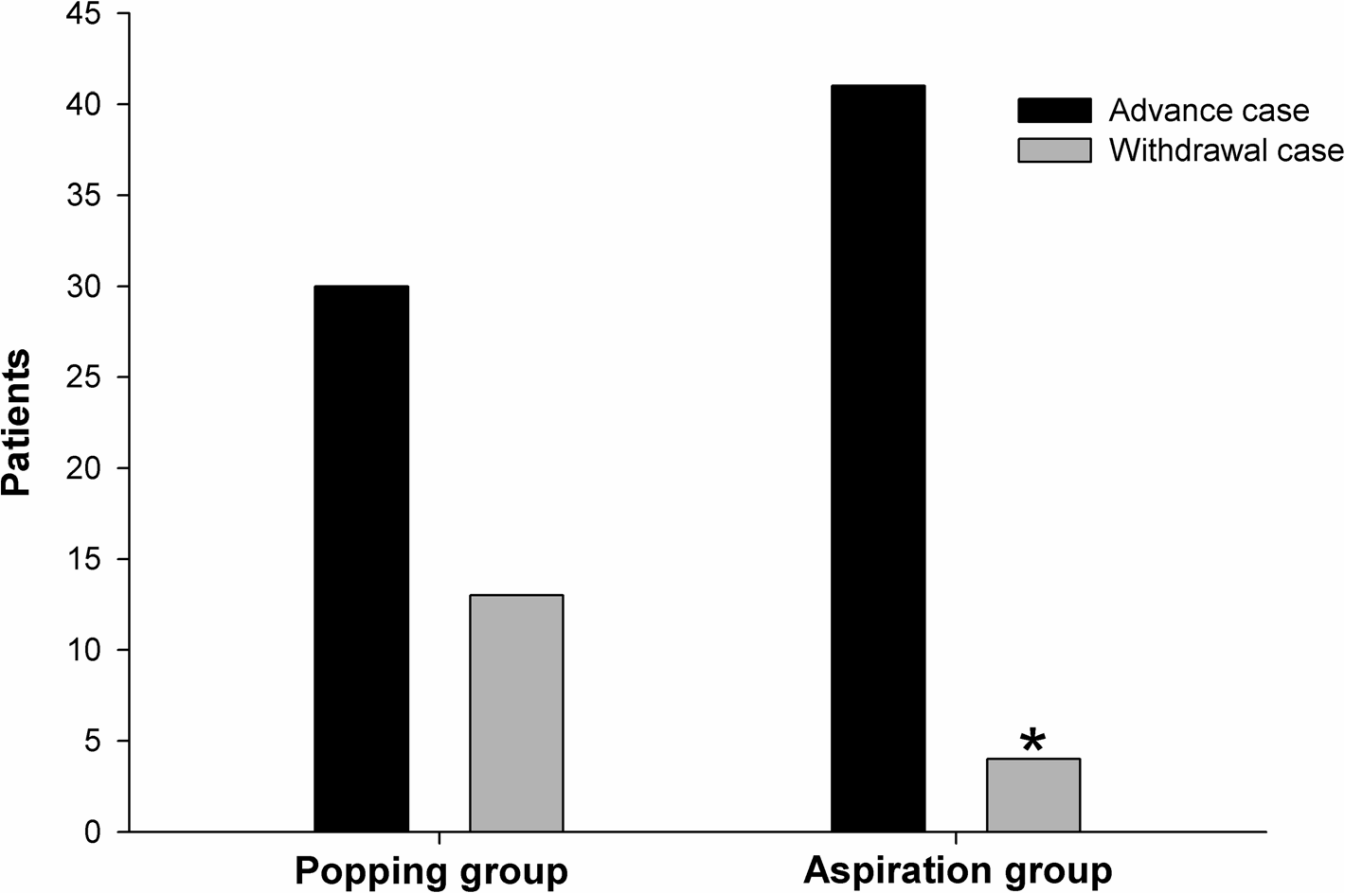
Withdrawal cases where cerebrospinal fluid was identified while withdrawing the needle in both groups. *Statistically significant (*P* ≤ 0.05)

**Fig. 4:**
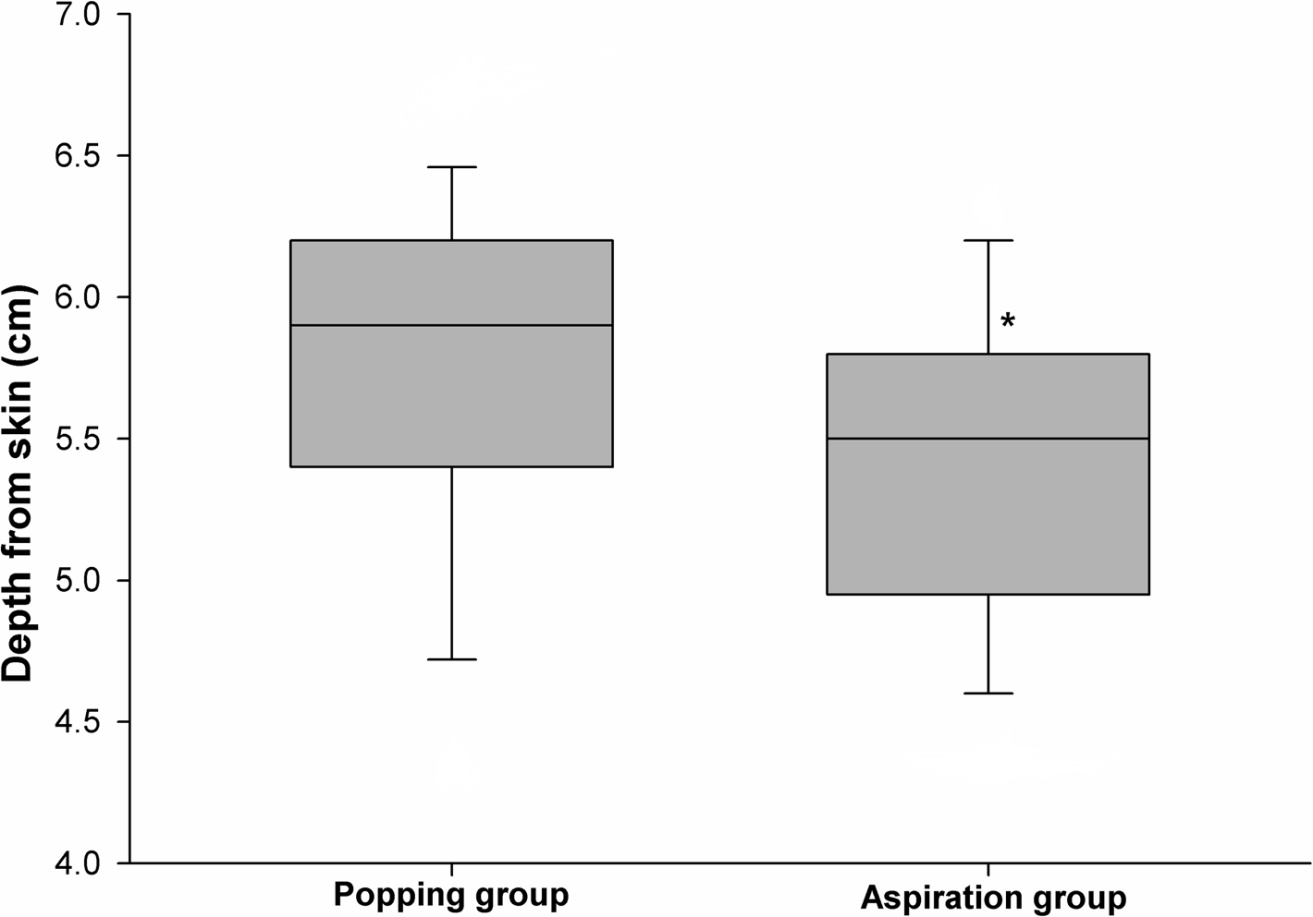
Actual depths of dural puncture in both groups. *Statistically significant (*P* ≤ 0.05)

Data related to dural puncture between the two groups are summarized in Table 2. Figure 2 shows the number of attempts for successful dural puncture in both groups. In the aspiration group, the success rate of the first attempt for dural puncture was 93.3% compared to 72.1% in the popping group (*P* = 0.019). The success rate of the first passage was 55.6% in the aspiration group and 34.9% in the popping group (*P* = 0.085). In the popping group, the number of attempts was significantly higher than in the aspiration group (*P* = 0.044). There was no difference in the number of needle passages between the two groups (*P* = 0.056). Figure 3 shows that withdrawal cases included four (8.9%) patients in the aspiration group and 13 (30.2%) in the popping group (*P* = 0.024). There was no difference in the successful attempt time between the two groups. In the popping group, the total procedure time was significantly longer (*P* = 0.023). Figure 4 shows the depths for actual dura puncture in the two groups. The actual depths of dural puncture were deeper in the popping group than in the aspiration group (*P* = 0.047).

**Table 2:** Profiles related to dural puncture between patients undergoing aspiration or conventional popping for spinal anesthesia

Variable	Dural puncture		*P* value
	Popping group (n = 43)	Aspiration group (n = 45)	
First attempt, success/failure cases, (n/n)	31/12	42/3	0.019*
First passage, success/failure cases, (n/n)	15/28	25/20	0.085
Number of passage (n)	2 (3)	1 (2)	0.064
Successful attempt time (s)	25 (52)	22 (19)	0.254
Total procedure time (s)	43 (109)	22 (20)	0.023*

Data are presented as raw count or median (interquartile range) unless otherwise indicated

The median (IQR) hyperbaric bupivacaine dose was 10.5 (1.88) mg in the popping group and 11 (1.25) mg in aspiration group (*P* = 0.071). The median anaesthesia level was T10 in both groups and was not significantly different (*P* = 0.364). No complications occurred in either group. The participants’ length of hospital stay was between 2 and 7 days; no patients presented with PDPH until discharge.

## Discussion

This prospective randomized study revealed that the aspiration method yields a significantly higher success rate during the first attempt, lower incidence of withdrawal, and lower number of attempts, along with shorter total procedure time, compared with the conventional popping method for spinal anaesthesia.

Performing spinal anaesthesia using a 27G Quincke needle may be difficult because the CSF pressure varies from 9 to 28 cmH_2_O, even in normal adults [10]. Even if a dural click is observed, CSF flow may be insufficient in a spinal needle with a thin diameter of 27G in situations involving low pressure. The dural puncture can be repeated without confirming the subarachnoid space only after spontaneous CSF flow is confirmed [11]. In this study, a higher first attempt success rate was noted in the aspiration group. The use of the aspiration method can help the operator detect the precise time of the dural puncture.

The number of attempts and total procedure time were significantly lower in the aspiration group. These favourable results suggest that the aspiration method can reduce complications arising from repeated manipulation of the spinal needle. Repeated attempts can directly damage the spinal cord or nerves of the central nervous system. Irritation of the filum terminale of the spinal cord causes paraesthesia, and in severe cases, nerve damage [12, 13]. There have been reports of damage to the conus medullaris [14] or discitis [15] after spinal anaesthesia. Considering the possibility of these complications, it may be inappropriate to perform spinal anaesthesia using the conventional popping method for advancing a spinal needle based on subjective feeling of the anaesthesiologist.

The aspiration method group showed favourable results in terms of success rate, likely because the operator easily identified the moment of dural puncture and CSF flow based on the results of withdrawal and puncture depth. In the conventional popping group, there were more withdrawal cases, in which the CSF flow was identified during backward movement of the needle. We speculate that this difference was because the anaesthesiologist using the conventional popping method overlooked the point of dural puncture during advancement of the needle, as there is no objective clue for dural puncture. The actual depth of the dural puncture was significantly deeper using the conventional popping method. The aspiration method may reduce the side effects caused by unnecessary deep penetration of the spinal needle.

This study compared the two methods for using the 27G Quincke spinal needle, which has a sharp cutting edge. While thinner spinal needles have previously been used, the thinnest needle currently used in clinical practice is 27G. The aim of using a thinner spinal needle is to reduce the incidence of PDPH or back pain. However, if the dural click is not identified, repeated dural punctures are required, and the procedure time is prolonged. This leads to an increased risk of patient discomfort, PDPH, back pain, mechanical damage of the nerve, and infection [16, 17]. Therefore, the use of an aspiration method is appropriate when performing spinal anaesthesia with a thin needle.

The depth of dural puncture was significantly deeper in the popping group. The depth difference was only 3 mm; thus, its value could be underestimated. However, it corresponded to 30% of the total subarachnoid space; the distance between anterior and posterior dura in this study was approximately 10 mm, a difference that should not be ignored. Spinal needles that are further advanced by 3 mm can penetrate the anterior dural mater through the subarachnoid space, increasing the possibility of damage to surrounding tissues. In addition, because the length of the needle bevel is 1.5 mm, the theoretical maximum difference between the two groups is 13 mm. In this study, a 3-mm difference in dural puncture is clinically relevant.

In this study, the measured distances from skin to the posterior and anterior dura complex were obtained from the lumbar spine lateral view. There were no differences in distances from the skin to the posterior and anterior dura complex on lumbar lateral X-ray film, as there were in other demographic characteristics of the patients. The distances from skin to the posterior and anterior dura complex are longer when compared to the actual depth of dural puncture. As the discrepancy is due to the patient’s position, this comparison would be desirable if distances were measured in the foetal posture. This can serve as a guide to avoid advancing deeper than the depth when using the lumbar spine lateral view. Further studies are needed to provide reliable guidelines for safe spinal anaesthesia.

Notably, if a pencil-point needle is available, the results of this study may not be useful. Regarding the type of needle, a 25G pencil-point spinal needle causes PDPH similar to a cutting type spinal needle of less than 27G [18]. Furthermore, when using the pencil-point needle, dural clicks are easy to recognize [19]. Based on the actual cost of medical care, clinicians should use 27G Quincke-type spinal needles in most situations. In order to avoid the challenges faced using the 27G needle that are described above, we have attempted the aspiration method; the results of this study reveal that the aspiration method can be more effective and safer than the conventional popping method.

### Limitations

There were some limitations to this study. First, there may be a concern about advancing the spinal needle without the stylet. If the needle passes the tissue without the stylet, a blood clot or tissue debris can occlude the needle. To minimize this problem, the operator used an introducing needle of 22G and removed the stylet when the needle entered the ligamentum flavum and proceeded with aspiration using the syringe. However, the stylet is not usually inserted when using the 27G needle in other procedure such as injection into the tissue. Second, the operator and investigator who watched the operator’s procedure and evaluated and recorded the data were not blinded to the patient groups. Finally, all subjects were aged < 65 years and had no spinal disorders or history of spinal surgery. Thus, our results may not necessarily be applicable to different patient populations, particularly those of an older age with degenerative changes, obesity, or spinal diseases such as scoliosis.

## Conclusions

The aspiration method has several clinical benefits for dural puncture compared with the conventional popping method for spinal anaesthesia that uses 27G Quincke-type needles.

## Data Availability

The data are not available for public access because of patient privacy concerns, but are available from the corresponding author on reasonable request.

## Abbreviations

27G: 27-gauge
CSF: Cerebrospinal fluid
IQR: Interquartile range
PDPH: Post-dural puncture headache
